# Supplementary material for: Inulin promotes appetite in mice by regulating the gut microbiota under conditions of rapid entry to the plateau
Source: PLoS One. 2025 Apr 24;20(4):e0322059. doi: 10.1371/journal.pone.0322059 (PMC12021179; doi:10.1371/journal.pone.0322059)
Supplement: S1 File — Raw gels from agarose gel electrophoresis of PCR products from feces of inulin-treated mice. (PDF) [file pone.0322059.s001.pdf]

美吉生物PCR扩增报告

1. 项目基本信息

项目名称	MJ20221008005-MJ-M-20221007027-陈潇楠-20221025PCR正式实验报告	项目跟进销售	张文君
项目联系人	陈潇楠	合同编号	MJ20221008005
收样人		收样日期	2022-10-12
PCR操作员	刘亚茹	PCR报告日期	2022-10-25
报告撰写人	刘亚茹	报告审核人	何灿

2. PCR预实验

2.1 引物设计

测序区域	引物名称	引物序列	备注
338F_806R	338F	ACTCCTACGGGAGGCAGCAG	
	806R	GGACTACHVGGGTWTCTAAT	

2.2 PCR扩增条件

为了保证后续数据分析的准确性及可靠性，需要两个条件，首先是尽可能使用低循环数扩增；其次保证每个样品扩增的循环数统一。为了达到以上目的，我方首先随机选取样品进行预实验，以确保在最低的循环数中绝大多数的样品能够扩增出浓度合适的产物，为所有样品的正式试验做充分准备。

在预实验完成后，PCR 正式试验采用TransGen AP221-02：TransStart Fastpfu DNA Polymerase，20μl反应体系：

5×FastPfu Buffer .... 4 μl

2.5 mM dNTPs .... 2 μl

Forward Primer(5 μM) .... 0.8 μl

Reverse Primer(5 μM) .... 0.8 μl

FastPfu Polymerase .... 0.4 μl

BSA .... 0.2 μl

Template DNA .... 10 ng

补ddH2O至 .... 20 μl

PCR仪：ABI GeneAmp® 9700型

PCR反应参数：

a. 1× (3 minutes at 95℃)

b. **循环数** × (30 seconds at 95℃；30 seconds at **退火温度** ℃；45 seconds at 72℃)

c. 10 minutes at 72℃，10℃ until halted by user

2.3 PCR扩增结果鉴定胶图

2%琼脂糖凝胶电泳检测PCR产物，3μl上样检测电泳图：

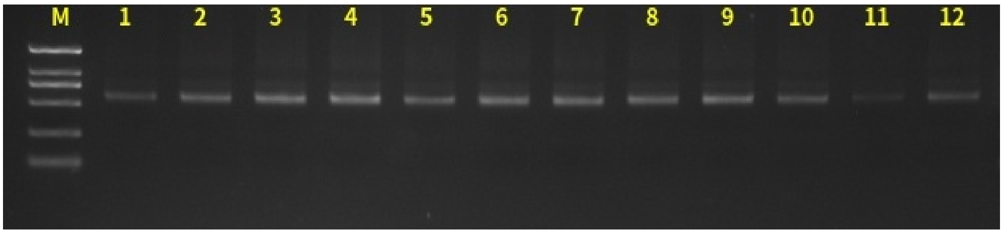

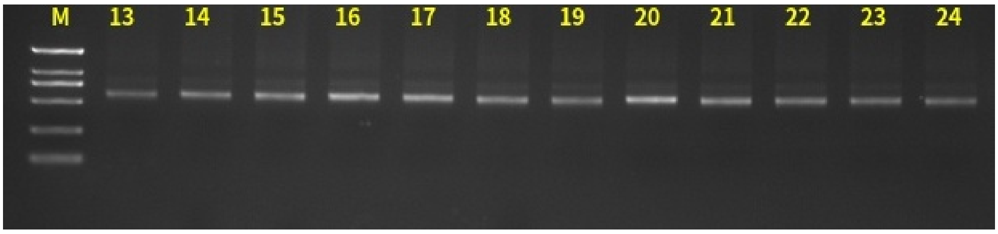

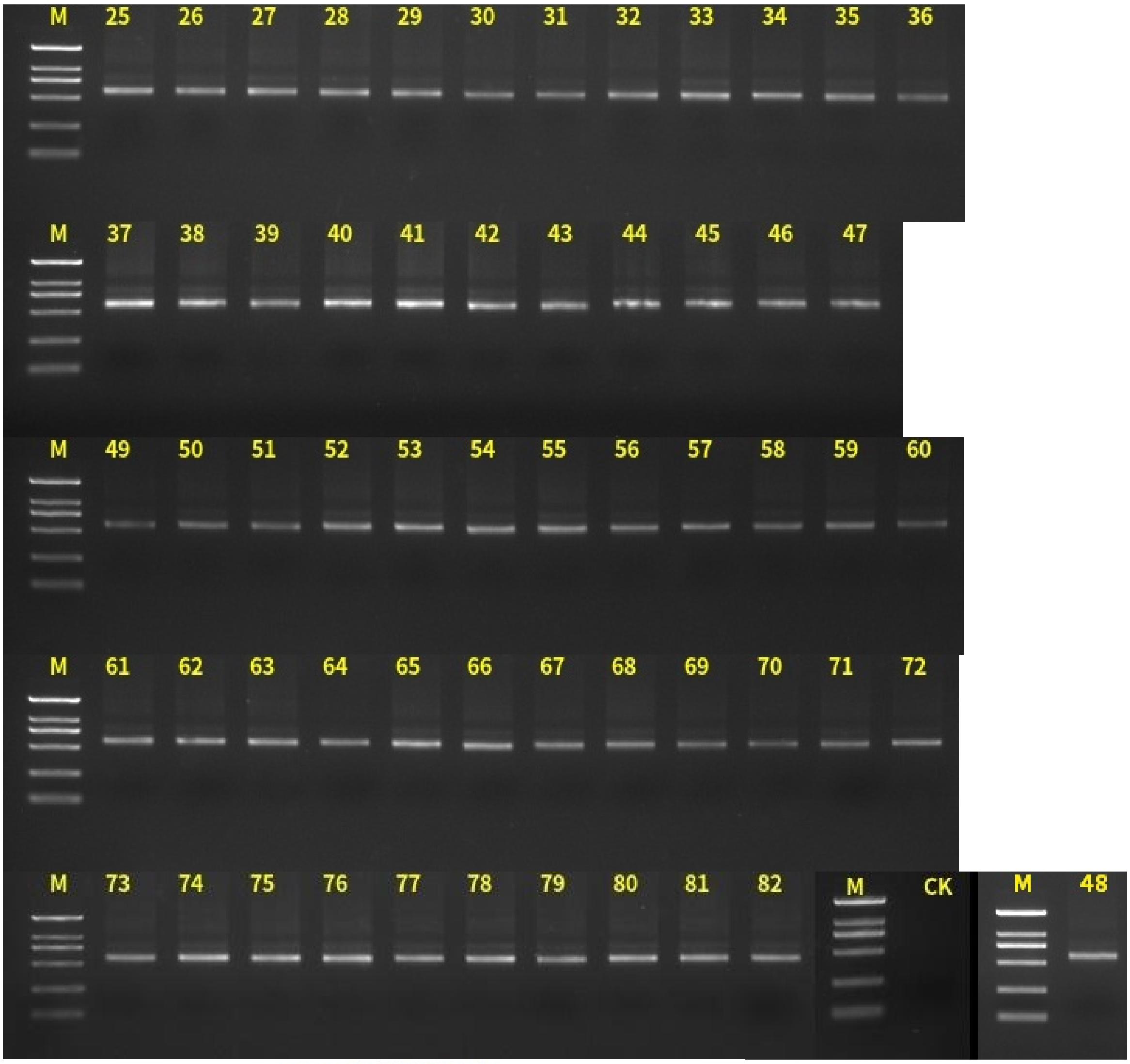

DL2000条带分布如下：

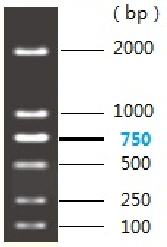

2.4 PCR扩增结果

我方已将贵方样品进行PCR扩增条件摸索，截止目前的PCR扩增结果统计如下：

胶图编号	样品名称	美吉编号	实验名称	测序引物	退火温度(℃)	循环数	结果
1	B1_1	EGJ1202021	陈潇楠A74	338F_806R	55	25	A
2	B1_2	EGJ1202022	陈潇楠A75	338F_806R	55	25	A
3	B1_3	EGJ1202023	陈潇楠A76	338F_806R	55	25	A
4	B1_4	EGJ1202024	陈潇楠A77	338F_806R	55	25	A
5	B1_5	EGJ1202025	陈潇楠A78	338F_806R	55	25	A
6	B2_1	EGJ1202026	陈潇楠A79	338F_806R	55	25	A
7	B2_2	EGJ1202027	陈潇楠A80	338F_806R	55	25	A
8	B2_3	EGJ1202028	陈潇楠A81	338F_806R	55	25	A
9	B2_4	EGJ1202029	陈潇楠A82	338F_806R	55	25	A
10	B2_5	EGJ1202030	陈潇楠A83	338F_806R	55	25	A
11	B3_1	EGJ1202031	陈潇楠A84	338F_806R	55	25	A
12	B3_2	EGJ1202032	陈潇楠A85	338F_806R	55	25	A
13	B3_3	EGJ1202033	陈潇楠A86	338F_806R	55	25	A

14	B3_4	EGJ1202034	陈潇楠A87	338F_806R	55	25	A
15	B3_5	EGJ1202035	陈潇楠A88	338F_806R	55	25	A
16	B4_1	EGJ1202036	陈潇楠A89	338F_806R	55	25	A
17	B4_2	EGJ1202037	陈潇楠A90	338F_806R	55	25	A
18	B4_3	EGJ1202038	陈潇楠A91	338F_806R	55	25	A
19	B4_4	EGJ1202039	陈潇楠A92	338F_806R	55	25	A
20	B4_5	EGJ1202040	陈潇楠A93	338F_806R	55	25	A
21	B5_1	EGJ1202041	陈潇楠A94	338F_806R	55	25	A
22	B5_2	EGJ1202042	陈潇楠A95	338F_806R	55	25	A
23	B5_3	EGJ1202043	陈潇楠A96	338F_806R	55	25	A
24	B5_4	EGJ1202044	陈潇楠A97	338F_806R	55	25	A
25	B5_5	EGJ1202045	陈潇楠A98	338F_806R	55	25	A
26	B6_1	EGJ1202046	陈潇楠A99	338F_806R	55	25	A
27	B6_2	EGJ1202047	陈潇楠A100	338F_806R	55	25	A
28	B6_3	EGJ1202048	陈潇楠A101	338F_806R	55	25	A
29	B6_4	EGJ1202049	陈潇楠A102	338F_806R	55	25	A
30	B6_5	EGJ1202050	陈潇楠A103	338F_806R	55	25	A
31	B7_1	EGJ1202051	陈潇楠A104	338F_806R	55	25	A
32	B7_2	EGJ1202052	陈潇楠A105	338F_806R	55	25	A
33	B7_3	EGJ1202053	陈潇楠A106	338F_806R	55	25	A
34	B7_4	EGJ1202054	陈潇楠A107	338F_806R	55	25	A
35	B8_1	EGJ1202055	陈潇楠A108	338F_806R	55	25	A
36	B8_2	EGJ1202056	陈潇楠A109	338F_806R	55	25	A
37	B8_3	EGJ1202057	陈潇楠A110	338F_806R	55	25	A
38	B8_4	EGJ1202058	陈潇楠A111	338F_806R	55	25	A
39	B8_5	EGJ1202059	陈潇楠A112	338F_806R	55	25	A
40	B9_1	EGJ1202060	陈潇楠A113	338F_806R	55	25	A
41	B9_2	EGJ1202061	陈潇楠A114	338F_806R	55	25	A
42	B9_3	EGJ1202062	陈潇楠A115	338F_806R	55	25	A
43	B9_4	EGJ1202063	陈潇楠A116	338F_806R	55	25	A
44	B9_5	EGJ1202064	陈潇楠A117	338F_806R	55	25	A
45	B10_1	EGJ1202065	陈潇楠A118	338F_806R	55	25	A
46	B10_2	EGJ1202066	陈潇楠A119	338F_806R	55	25	A
47	B10_3	EGJ1202067	陈潇楠A120	338F_806R	55	25	A
48	B10_4	EGJ1202068	陈潇楠A121	338F_806R	55	25	A
49	B10_5	EGJ1202069	陈潇楠A122	338F_806R	55	25	A
50	B11_1	EGJ1202070	陈潇楠A123	338F_806R	55	25	A
51	B11_2	EGJ1202071	陈潇楠A124	338F_806R	55	25	A
52	B11_3	EGJ1202072	陈潇楠A125	338F_806R	55	25	A
53	B11_4	EGJ1202073	陈潇楠A126	338F_806R	55	25	A
54	B11_5	EGJ1202074	陈潇楠A127	338F_806R	55	25	A
55	B11_6	EGJ1202075	陈潇楠A128	338F_806R	55	25	A
56	B11_7	EGJ1202076	陈潇楠A129	338F_806R	55	25	A
57	B1_a1	EGJ1202077	陈潇楠A130	338F_806R	55	25	A
58	B1_a2	EGJ1202078	陈潇楠A131	338F_806R	55	25	A
59	B1_a4	EGJ1202079	陈潇楠A132	338F_806R	55	25	A
60	B2_a1	EGJ1202080	陈潇楠A133	338F_806R	55	25	A
61	B2_a2	EGJ1202081	陈潇楠A134	338F_806R	55	25	A
62	B2_a4	EGJ1202082	陈潇楠A135	338F_806R	55	25	A
63	B2_a5	EGJ1202083	陈潇楠A136	338F_806R	55	25	A

64	B5_a1	EGJ1202084	陈潇楠A137	338F_806R	55	25	A
65	B5_a2	EGJ1202085	陈潇楠A138	338F_806R	55	25	A
66	B5_a3	EGJ1202086	陈潇楠A139	338F_806R	55	25	A
67	B5_a4	EGJ1202087	陈潇楠A140	338F_806R	55	25	A
68	B5_a5	EGJ1202088	陈潇楠A141	338F_806R	55	25	A
69	B6_a1	EGJ1202089	陈潇楠A142	338F_806R	55	25	A
70	B6_a2	EGJ1202090	陈潇楠A143	338F_806R	55	25	A
71	B6_a3	EGJ1202091	陈潇楠A144	338F_806R	55	25	A
72	B6_a4	EGJ1202092	陈潇楠A145	338F_806R	55	25	A
73	B6_a5	EGJ1202093	陈潇楠A146	338F_806R	55	25	A
74	B7_a1	EGJ1202094	陈潇楠A147	338F_806R	55	25	A
75	B7_a2	EGJ1202095	陈潇楠A148	338F_806R	55	25	A
76	B7_a3	EGJ1202096	陈潇楠A149	338F_806R	55	25	A
77	B7_a4	EGJ1202097	陈潇楠A150	338F_806R	55	25	A
78	B8_a1	EGJ1202098	陈潇楠A151	338F_806R	55	25	A
79	B8_a2	EGJ1202099	陈潇楠A152	338F_806R	55	25	A
80	B8_a3	EGJ1202100	陈潇楠A153	338F_806R	55	25	A
81	B8_a4	EGJ1202101	陈潇楠A154	338F_806R	55	25	A
82	B8_a5	EGJ1202102	陈潇楠A155	338F_806R	55	25	A

2.5 结果说明

结果代码	预实验结果说明
A	PCR产物目的条带大小正确，浓度合适，可进行后续实验；
B	PCR产物目的条带大小正确，浓度偏低，可尝试进行后续实验；
C	PCR产物目的条带太弱或未检测到，无法进行后续实验，需要重新提供样品；
