# Supplementary material for: Inulin promotes appetite in mice by regulating the gut microbiota under conditions of rapid entry to the plateau
Source: PLoS One. 2025 Apr 24;20(4):e0322059. doi: 10.1371/journal.pone.0322059 (PMC12021179; doi:10.1371/journal.pone.0322059)
Supplement: Attachment — Submitted filename: Report.pdf [file pone.0322059.s004.pdf]

Present work, titled as "*Inulin promotes appetite in mice by regulating the gut microbiota under conditions of rapid entry to the plateau*", by Xiaoli Li and co-workers aims to explore role of inulin, a dietary fiber, in regulating gut microbiota with a model study using fifty seven-week-old SPF-grade C57BL/6J male mice. Authors have chosen two different environmental conditions, i.e. regular and rush to plateau to study structural as well as functional characteristics of inulin uptake affecting the gut microbiota. Results demonstrate that inulin has a direct impact on metabolism and secretion of appetite hormones which result in enhanced appetite and body weight by increasing useful gut bacteria.

Authors have systematically introduced the concepts involved in developing the study of inulin in the context of plateau health supplements along with detailed methodology adopted in conducting the study. The manuscript is well-written, highlighting the background of current study, limitations/challenges followed by scope of future actions towards promoting health in plateau environments. The work can be accepted for publication after addressing the following concerns:

Correction:

- Authors mentioned in the abstract that 'fifty seven-week-old SPF-grade C57BL/6J male mice' were used for the study but materials and methods section says fifty eight week. Please check and correct this.
- What do NPY and PYY correspond to in the 'Measurement of appetite hormone content' section? Explain the significance of these measuring these two contents for better understanding of the readers.
- Which 'spectrophotometer' was used to measure the purity and concentration of extracted DNA? Provide the details.
- Provide the reference for 'four parameter logistic function'.
- Several sentences need to be rephrased for clarity like;
  'Overcoming the adverse effects..solved urgently.'
  'Furthermore, the mechanism...inulin was analyzed.'
  'Were/was' used at several places in the results and discussion section.
  'pH' written as 'PH'.
  'Inulin' should be written in lower case whenever it appears in the middle of the sentence.
