# Supplementary material for: Inulin promotes appetite in mice by regulating the gut microbiota under conditions of rapid entry to the plateau
Source: PLoS One. 2025 Apr 24;20(4):e0322059. doi: 10.1371/journal.pone.0322059 (PMC12021179; doi:10.1371/journal.pone.0322059)
Supplement: Attachment — Submitted filename: Response to Reviewers.docx [file pone.0322059.s005.docx]

*Dear Editor,*

I hope this message finds you well.

I am happy to inform you that I have revised my manuscript in line with your feedback. Please find below a list of the primary adjustments made:

The figure captions have been finished.

The data about this has been upload in form of supporting information.

A statistical analysis of the relative abundance of microbiota has been conducted using the FDR correction to address the issue of controlling the false discovery rate.

The manuscript's "Methods" section has been updated with additional information on ethical considerations regarding animal experimentation, euthanasia procedures, anaesthesia protocols, and efforts to minimise suffering.

A comprehensive data availability statement is included.

A caption has been included for each figure, presented separately.

The original, uncropped and unadjusted images that form the basis of the gel results have been submitted.

In response to the query about the housing of the mice and the number of cages used, the original text already provides this information in the Methods section. The animals were housed in five cages (n = 10 per cage) with wood shavings and fed and watered ad libitum.

I am grateful for your valuable review suggestions and kindly request a second review of the revised manuscript. I am open to receiving all your feedback and look forward to hearing from you at your earliest convenience.

Thank you once again.

Best regards,

*Li Xiaoli*
