# Supplementary material for: Inulin promotes appetite in mice by regulating the gut microbiota under conditions of rapid entry to the plateau
Source: PLoS One. 2025 Apr 24;20(4):e0322059. doi: 10.1371/journal.pone.0322059 (PMC12021179; doi:10.1371/journal.pone.0322059)
Supplement: Attachment — Submitted filename: Response_to_Reviewers_auresp_2.docx [file pone.0322059.s006.docx]

*Dear Editor and Reviewers,*

Thank you for your constructive feedback and careful evaluation of our manuscript titled “Inulin promotes appetite in mice by regulating the gut microbiota under conditions of rapid entry to the plateau.” We have thoroughly addressed all the concerns raised and incorporated the suggested revisions. Below are our point-by-point responses:

1. **Correction: Inconsistency in mouse age**

Reviewer’s comment: The abstract states “fifty seven-week-old mice,” while the Materials and Methods section mentions “fifty eight-week-old mice.”

**Response:** We sincerely apologize for this oversight. The correct age of the mice is seven-week-old, as stated in the abstract. The typographical error in the Materials and Methods section has been corrected (Page 3, Line 78).

2. **Clarification of NPY and PYY**

Reviewer’s comment: Explain the abbreviations NPY and PYY and their significance in appetite regulation.

**Response:** We have revised the “Measurement of appetite hormone content” section to clarify:

Neuropeptide Y(NPY, an appetite-stimulating hormone). Peptide YY(PYY, an appetite-suppressing hormone).This explanation has been added to the manuscript (Page 6, Lines 68–69).

Measuring these hormones helps elucidate how inulin modulates appetite via gut microbiota-mediated pathways. This explanation has been added to the manuscript (Page 6, Lines 112–114).

3. **Spectrophotometer details**

Reviewer’s comment: Specify the spectrophotometer used for DNA analysis.

**Response:** The purity and concentration of the extracted DNA were measured using an AOELAB V-1200 spectrophotometer made by Xiangyi Instruments Co. Ltd (Shanghai, China).

This detail has been included in the Methods section (Page 4, Line 73-76).

4. **Reference for the four-parameter logistic function**

Reviewer’s comment: Provide a reference for the four-parameter logistic function.

**Response:** We have added the requested reference (DeLean et al., 1978) as citation [27] in the revised manuscript (Page 9, Line 191).

**5. Language and formatting revisions**

Reviewer’s comment: Rephrase ambiguous sentences, correct grammatical errors, and standardize formatting (e.g., “pH,” lowercase “inulin”).

**Response:** All suggested revisions have been implemented:

Ambiguous sentences (e.g., “Overcoming the adverse effects…solved urgently”) were rephrased for clarity.

Grammatical inconsistencies (e.g., “were/was”) and formatting errors (e.g., “PH” → “pH”) were corrected.

“Inulin” is now consistently lowercase in mid-sentence instances.

We appreciate the reviewer’s meticulous attention to these details.

**Additional Changes**

All figures, tables, and supplementary materials were reviewed for consistency, and minor typographical errors were corrected throughout the manuscript.

We believe these revisions have significantly strengthened the manuscript. Thank you again for your valuable feedback. Please let us know if further clarifications or adjustments are required.

Sincerely,
Xiaoli Li (on behalf of all authors)
